# A Comprehensive Review for Groundwater Contamination and Remediation: Occurrence, Migration and Adsorption Modelling

**DOI:** 10.3390/molecules26195913

**Published:** 2021-09-29

**Authors:** Osamah Al-Hashimi, Khalid Hashim, Edward Loffill, Tina Marolt Čebašek, Ismini Nakouti, Ayad A. H. Faisal, Nadhir Al-Ansari

**Affiliations:** 1 Babylon Water Directorate, Babylon 51001, Iraq; 2 School of Civil Engineering and Built Environment, Liverpool John Moores University, Liverpool L3 3AF, UK; K.S.Hashim@ljmu.ac.uk (K.H.); E.Loffill@ljmu.ac.uk (E.L.); T.MaroltCebasek@ljmu.ac.uk (T.M.Č.); 3 Department of Environmental Engineering, College of Engineering, University of Babylon, Babylon 51001, Iraq; 4 Built Environment and Sustainable Technology Research Institute, Liverpool John Moores University, Byrom Street, Liverpool L3 3AF, UK; I.Nakouti@ljmu.ac.uk; 5 Department of Environmental Engineering, College of Engineering, University of Baghdad, Baghdad 10001, Iraq; ayadabedalhamzafaisal@yahoo.com; 6 Department of Civil, Environmental and Natural Resources Engineering, Lulea University of Technology, 97187 Lulea, Sweden; nadhir.alansari@ltu.se

**Keywords:** adsorption, groundwater, remediation, isotherm, breakthrough curve, permeable reactive barrier, sorption models

## Abstract

The provision of safe water for people is a human right; historically, a major number of people depend on groundwater as a source of water for their needs, such as agricultural, industrial or human activities. Water resources have recently been affected by organic and/or inorganic contaminants as a result of population growth and increased anthropogenic activity, soil leaching and pollution. Water resource remediation has become a serious environmental concern, since it has a direct impact on many aspects of people’s lives. For decades, the pump-and-treat method has been considered the predominant treatment process for the remediation of contaminated groundwater with organic and inorganic contaminants. On the other side, this technique missed sustainability and the new concept of using renewable energy. Permeable reactive barriers (PRBs) have been implemented as an alternative to conventional pump-and-treat systems for remediating polluted groundwater because of their effectiveness and ease of implementation. In this paper, a review of the importance of groundwater, contamination and biological, physical as well as chemical remediation techniques have been discussed. In this review, the principles of the permeable reactive barrier’s use as a remediation technique have been introduced along with commonly used reactive materials and the recent applications of the permeable reactive barrier in the remediation of different contaminants, such as heavy metals, chlorinated solvents and pesticides. This paper also discusses the characteristics of reactive media and contaminants’ uptake mechanisms. Finally, remediation isotherms, the breakthrough curves and kinetic sorption models are also being presented. It has been found that groundwater could be contaminated by different pollutants and must be remediated to fit human, agricultural and industrial needs. The PRB technique is an efficient treatment process that is an inexpensive alternative for the pump-and-treat procedure and represents a promising technique to treat groundwater pollution.

## 1. Introduction

Earth is known as the blue planet or the water planet because of the reality that most of its surface is covered by water, and it is the only planet in the solar system that has this huge quantity of water [1,2]. For various authorities and agencies dealing with water problems, the conservation of surface and groundwater purity without pollution is indeed an aim. In addition, groundwater is the main potable water supply used in many nations; this is also water for agriculture and industry [3,4]. The effect of global warming, climate change, the rise in weather temperature and evaporation increment, population growth, excessive use of fresh water in agriculture and industrial activities have all led to increasing reliance on groundwater [5,6]. Groundwater became fundamental for social and economic development. It is the sole source for drinking to about 2.5 billion people around the world [7]. There are many reasons to develop groundwater, but among the most important are [8]:

(1) Groundwater usually lies in underground natural reservoirs. This promotes groundwater as a convenient source of water. Additionally, groundwater can be found in different quantities depending on aquifer capacity. Many times, aquifers detaining water larger than many human-made reservoirs; for example, the Ogalalla aquifer located in the United States produced up to 500 Km^3^ of water for four decades, which is larger than Nasser lake in Egypt. The huge quantities of groundwater give an ability to pump water during the drought period, while surface water (in some places) is unable to be pumped in these quantities or at such high quality during such period.
(2) In many cases, groundwater quality is better than surface water. This is due to the ability of aquifers to provide natural protection for groundwater from contamination.
(3) Groundwater is a cheap, reliable source of water. It can be pumped out using small capital and can be drilled close to the location needed for water. Additionally, groundwater can be easily organized, managed and developed. For example, individuals can easily construct and operate their groundwater well on their land.

Pumping and treatment is a common technique used for groundwater treatment; however, the lack of groundwater quality restoration in the long term has been demonstrated in this method. An innovative approach to groundwater remediation is, therefore, necessary. The permeable reactive barrier (PRB) is proven as a promising technology for groundwater treatment by an interaction between the reactive material and the contaminant when the dissolved compounds migrate. In the permeable reactive barrier (PRB), water moves in a natural gradient, and no further energy is used to achieve the treatment [9]. The PRB is classified as in situ treatment, and the contaminant is transformed in the contaminated site into less toxic or immovable forms. The key benefits of the PRB innovation are minimal maintenance costs and long durability. However, the aim of this work is that future researchers will find a clear, in-depth and detailed explanation of groundwater contaminants, movement and detailed theoretical explanation for the fate of contaminants in the environment.

## 2. Groundwater Contamination

Groundwater is the global population’s main source of fresh water and is used for domestic, food production and industrial purposes. About a third of the world’s population depends on groundwater as the main water source for their drinking purposes [10]. According to the United Nations Environmental Agency (UNEP), there are 32 cities around the world with a population greater than 10 million known as “megacities”; about 16 of these cities majorly rely upon groundwater [8]. In China, there are 657 cities, and approximately 400 cities are using water from the ground as the main source for their water supplies [11]. It is without doubt that subsurface water/groundwater is an essential resource of water to humanity; furthermore, it is vital for the ecological system on earth. Keeping this water resource sustainable, accessible, effective and efficient is a major concern for scientists working in a related field. However, urbanization, farming, industry and climate change all pose significant threats to the quality of groundwater. Toxic metal, hydrocarbons, contaminants such as organic trace pollutants, pharmaceutical pollutants, pesticides and other contaminants are endangering human health, natural ecosystems and long-term socioeconomic development [12,13]. Chemical contamination has been a major subject in groundwater investigations in recent decades. While groundwater contamination poses a significant threat to human populations, it also provides a chance for researchers to learn more about how our underground aquifers have evolved, as well as for decision makers to understand how we might maintain the quality and quantity of these resources [14]. According to the Canadian government, the contamination of groundwater can be defined as the addition of undesired substances by human activities [15]. Chemicals, brines, microbes, viral infections, medications, fertilizers and petroleum can all contribute to groundwater contamination. However, groundwater contamination is differs from surface water contamination in that it is unseen, and recovery of the resource is difficult and expensive at the current technological level [16].

Due to human and natural activities, chemicals and pollutants may be found in groundwater. Metals such as arsenic, cadmium and iron could be dissolved in groundwater and may be found in high concentrations. Human activities such as industrial discharges, waste disposal and agriculture activities are the main cause of groundwater contamination. Furthermore, it could happen due to urban activities such as the excessive use of fertilizers, pesticides and chemicals in which pollutants migrate to groundwater and reach the water table. In any case, using groundwater for drinking, irrigation or industrial purposes requires different tests to ensure that it is suitable for these purposes.

The presence of inorganic contaminants in groundwater is a big concern especially when groundwater is used for drinking or agricultural purposes. If these contaminants are presented in the groundwater with levels higher than the permissible recommended concentration, they cause health problems throughout the food chain [17]. Table 1 presents different inorganic pollutants in groundwater, sources and health effects.

In addition, discharging organic pollutants into the environment and water resources represents a pressing concern for people’s health. The existence of organic contaminants in groundwater represents a crucial environmental problem, as it may affect the water supply reservoirs and people’s health [18]. Additionally, it can affect the ecological system [19]. Usually, groundwater contaminants come from two sources: (1) landfills, solid waste disposal lands, sewer leakage and storage tanks leakage and (2) agriculture and farmyard drainage [20]. Table 2 shows the most common organic pollutants usually found in the groundwater, the sources and the health effect.

In the environment, groundwater in shallow or deep aquifers is never found completely sterile [21]. Coliform organisms and bacteria are the main cause of the microbiological pollution of groundwater. When present, these pollutants need immediate attention to protect lives from outbreaks of pathogenic disease [22]. Microbiological contaminants naturally occur in the environment by the intestines of humans, warm-blooded animals and plants. These microorganisms could cause dysentery, typhoid fever and different diseases [21].

## 3. Groundwater Treatment Technologies

In recent decades, scientists developed sophisticated and highly successful techniques for the remediation of water from many contaminants. These techniques generally focused on the treatment of surface water resources such as a river, lakes and water reservoirs. However, in recent years, scientists and environmental researchers have become more aware of treating underground water, and groundwater has become an essential source of water in most places; it represents about 30% of the freshwater reserve in the world [29,32,37,38]. Groundwater is usually treated by drilling water wells, pumping the polluted water to ground facilities to perform different approaches of treatment such as air stripping and treatment tower and granular activated carbon (GAC). Pressurized air bubbles are also used to treat contaminated groundwater. The selection of the effective treatment/remediation procedure depends on the characteristics of contaminants and pollutants, in addition to the reactive media available [39].

### 3.1. Pump and Treat Method

One of the popular procedures to remediate contaminated groundwater is by dissolved chemicals, solvents, metals and fuel oil [40]. In this procedure, contaminated groundwater is piped to ground lagoons or directly to treatment units, which treat the groundwater using various methods such as activated carbon or air stripping. Finally, the treated water is to be discharged either to the nearest sewer system or re-pumped to the subsurface [37]. This technique can treat large volumes of contaminated groundwater but has many disadvantages, such as the high cost, spreading of contaminants into the ecosystem, as well as its long operation time; in addition, it may cause a reversal to the hydraulic gradient [41,42,43] as cited in [40].

### 3.2. Air Sparging Procedure and Soil Vapor Extraction

The procedure of air sparging and soil vapor extraction (SVE) is considered one of the most common techniques used in remediating groundwater contaminated by volatile organic contaminants (VOCs). It is considered efficient, fast and relatively economical [44]. This method involves the injection of pressurized air at the lowest point of the contaminated groundwater; this will clean up the groundwater by changing the state of volatile hydrocarbons to a vapor state. While pumping air under the saturated zone, pollutants are stripped out of the aquifer and oxygen is provided for the biodegradation of contaminants [45]. The extracted air is to be treated by vacuum extraction systems to remove any toxic contaminants [46]. The limitations for this method are the high cost when working in hard surface area and when many deep wells are required for the treatment. In addition, soil heterogeneity may lead to uneven treatment of the contaminated groundwater.

### 3.3. The Permeable Reactive Barriers (PRBs)

It is an innovative remediation technique [47]. Practically, it is in situ technology to remediate groundwater using reactive media designed to intercept a contaminated plume. Typically, reactive media is designed to degrade volatile organics, immobilizing metals. PRB media is placed with porous materials such as sand; this will enhance the hydraulic conductivity, so the plume of contaminants will pass through the PRB under a natural gradient descent [37,48].

In the treatment wall, contaminants are removed by adsorbing, transforming, degrading and precipitating the targeted pollutants during water flow through barrier trenches. PRBs are defined as an in situ remediation zone in which contaminants are passively captured, removed or broken down while it allows uncontaminated water to pass through. The primary removal method is either physical (sorption, precipitation), chemical (ion exchange) or biological [49,50,51,52].

There are many geometries for placing the permeable reactive barriers (PRBs): (1) A continuous wall that contains reactive media. This is the most common placement in which the reactive media is placed perpendicular to the contaminated plume of groundwater flow; (2) funnel and gate in which contaminant plume is directed to a treatment filtering gate by two-sided impermeable walls at sites in which the soil is very heterogeneous, placing the PRB in the most permeable portion of the soil. Furthermore, when the contaminant’s distribution is non-uniform, the pollutant’s concentration can be better homogenized when entering the PRB gate; (3) radial filtration/caisson configuration in which the filter is placed in a cylindrical shape of reactive media surrounded by coarse material with a core of course materials. Additionally, there must be a radial centripetal flow by applying a hydraulic gradient. The third type of PRB has a long lifespan and a better treatment efficiency by extending the contact time between the pollutant and the reactive barrier [47,53,54].

Different reactive materials can be used to remediate contaminants, for example, zero-valent iron (ZVI; Fe0), which is a mild reductant and can treat heavy metals. ZVI can de-halogenate may halogenate hydrocarbon derivatives [55]. Bio-sparging materials and slow oxygen releasing compounds have the ability to treat groundwater containing petroleum hydrocarbon plums such as nitrobenzene and aniline by utilizing the biodegradation of these pollutants in PRBs [56]. Vegetative materials could be used in PRBs such as mulch to remediate chlorinated solvents and perchlorates [57].

Contaminants can also be precipitated on chemical reactive materials in the PRBs, for example, fly ash, ferrous slats, lime, phosphates and zeolites, iron/sand, iron/gravel, iron/sponge, granular activated carbon, organic carbon, copper wool and steel wool [37,54].

#### 3.3.1. Characteristics of the Reactive Medias

Choosing a good reactive media depends on the following characteristics [58]:

1. Reactivity: The ability of reactive media to react/remediate contaminants and the equilibrium constant. All these factors are necessary to determine the required time for the remediation, which is important to calculate the volume and size of the in situ reactive barriers.
2. Stability: It is required that any good reactive material is to be active for a long period to remediate groundwater. Additionally, it is also necessary that the reactive media stay under the surface as a secondary precipitate. Once the PRB is installed, it is very expensive to be excavated and replaced with a new PRB.
3. Cost and availability: it is very important that the reactive media be available and inexpensive.
4. Hydraulic conductivity: the PRB must have a permeability equal to or greater than the surrounding soil to ease the groundwater flow within the PRB and achieve the remediation.
5. Environmental compatibility: Reactive media need to be similar/match the surrounding subsurface soil by mean of grain size for the goal that there will be no change in the hydraulic conductivity of the soil. Additionally, it needs no unwanted by-products to be produced during the remediation.

#### 3.3.2. Uptake Mechanism of Contaminants

In the remediation of groundwater from contaminants, four physical, chemical and biological uptake mechanisms are considered as uptake mechanisms [58,59,60], which are: (1) adsorption and ion exchange, (2) abiotic redaction, (3) biotic reduction and (4) chemical precipitation. Remediation of contaminants in groundwater can be achieved by two or more of these mechanisms [61].

(1) Adsorption and Ion Exchange

The process in which species in an aqueous environment are attached to a solid surface is referred to as adsorption. Usually, adsorption interaction is considered a rapid and reversible phenomenon. Adsorbents such as zero-valent iron (ZVI), zeolite and amorphous ferric oxyhydroxide (AFO) are the most common adsorbents used in the adsorption of contaminants; most of the adsorbents have a large surface area per gram and could be used in a PRB. ZVI has the most adsorption rate, and it is the most popular reactive media used in PRBs. Adhesion of pollutant’s ions, atoms or molecules while it is in a liquid, gas or dissolved solid state is referred to as adsorption. It utilizes chemical forces to create a thin film of the adsorbate on the adsorbent’s surface. The adsorbent is any kind of material that can adsorb substances through its surface area characteristics. In the adsorption theory, the surface area of the adsorbent is predominant. The solid phase that provides a working adsorption area is the adsorbent, while the substances and species adsorbed on the adsorbent are referred to as the adsorbate. Adsorption efficiency depends on adsorbate concentration, liquid-phase temperature and pH [62].

Ion exchange is a process of remediation of inorganic chemicals and dissolved metals from liquids and groundwater. The ion exchange process is that the ion (a single atom or group of atoms) is either positively charged after its loss of electrons or negatively charged after gaining an electron. When liquids loaded by pollutants pass through the ion exchange resin, contaminated substances will be exchanged by the effect of metallic ions attraction by the resins. These resins can be re-generated after being exhausted, or it may be a single-use resin [63,64]. Ion exchange phenomena is a reversible reaction process in which a pollutant’s ion is replaced with an identical ion on the immobilizing barrier. Most ion exchangers are natural such as zeolite, but also, there are very good synthesized ion exchanger resins that can be used in specific needs, especially for the treatment of inorganic contaminants [58,60]. The ion exchange method is applicable to remediate heavy metals [65] and dissolved metals (chromium) from polluted liquids. Additionally, this method could be used to treat non-metallic pollutants such as nitrate and ammonia [63]. The limitation to the use of this method is that the oxidation of the soil will cause damage to the resin and will decrease remediation efficiency [66,67]. Another concern is that the contaminant has not been destroyed if treated by the ion exchange method; it is only transferred to another medium that needs to be disposed of. This method is not good if the groundwater contains oil or grease, as these pollutants may clog the exchange resin [67].

(2) Abiotic Reduction

The chemical reactions that lead to the decomposition of contaminants in groundwater are referred to as abiotic remediation. In this technique, the harmful compounds are to be reduced either by immobilization in the treatment wall of the reactive barriers, or it is permitted to pass through the barrier in a harmless form. Zero-valent iron (ZVI) is the most popular reactive material used in the abiotic remediation of groundwater; after the reaction of ZVI with the contaminants, low solubility minerals will be precipitated, for example, the remediation of U and Cr from groundwater, which is removed by the precipitation of these contaminants by the abiotic process. Equation (1) shows the ability of ZVI to reduce U(VI) to U(IV) in groundwater with high carbonate and moderate pH via producing UO^2^ (Uraninite), which is a solid, less crystalline product of uranium.

(1) $$Fe^{0}\left[ZVI\right]+UO_{2}{\left(CO_{3}\right)}_{2}^{2-}+2H^{+}=UO_{2\left[Solid\right]}+2HCO_{3}^{-}+Fe^{2+}$$

For the chromium (Cr), ZVI reducing Cr(VI) to Cr(IV) [58,60] as shown in Equation (2):

(2) $${\mathrm{Fe}}^{0}\left[\mathrm{ZVI}\right]+8H^{+}+{\mathrm{CrO}}_{4}^{2-}={\mathrm{Fe}}^{3+}+{\mathrm{Cr}\;}^{3+}+4H_{2}O$$

Cr(VI) could be reduced to Cr(III) by ferrous iron via introducing dissolved dithionite ions ($S_{2}O_{4}^{2-}$) to an aquifer, which can reduce the solid phase of ferric iron. Dithionite oxidizes to sulphite ($SO_{3}^{2-}$) and $Fe^{3+}$ is lowered to $Fe^{2+}$. Cr(III) is to be stalemated by precipitate in the solid form of Cr(III) and Fe(III) hydroxide along with the reduction in some halogenated organic compounds by the effect of $Fe^{2+}$ as shown in Equations (3) and (4).

(3) $$S_{2}O_{4}^{2-}+2\;Fe{\left(III\right)}_{\left[Solid\right]}+2H_{2}O=2SO_{3}^{2-}+2Fe{\left(II\right)}_{\left[Solid\right]}+4H^{+}$$

(4) $$C_{r}O_{4}^{2-}+3\;Fe{\left(II\right)}_{\left[Solid\right]}+5H^{+}=Cr{\left(OH\right)}_{3\left[Solid\right]}+3Fe{\left(III\right)}_{\left[Solid\right]}+H_{2}O$$

(3) Biotic Redaction/Oxidation

When physical or chemical remediation of groundwater shows little or no degradation of contaminants, then degrading pollutants with a biological oxidation process may be helpful. Many pollutants such as chlorinated solvents tend to be easily reduced if oxidized; here, microorganisms will perform a reduction process by exploiting contaminants as their main source for energy and the required materials to synthesize their cells [49]. The bioremediation technique is a very effective remediation process based upon the degradation of contaminants by microorganisms; remediation efficiency in this process depends on the working environment, such as the temperature, pH, electron acceptors and the concentration of nutrients [68]. In biodegradation, it is necessary that germs use electron acceptors to accept any electrons liberated from pollutants; electrons transfer, releasing energy that is essential for microbes’ lives. In the presence of oxygen, under aerobic conditions (which is preferable), energy producing from this process is higher than that released without the presence of oxygen. Additionally, the oxidation rate of contaminants is higher. In the groundwater, the presence of oxygen is usually little; in this case, the anaerobic microbes electron acceptors is utilized. However, it is effective to remediate groundwater contaminated by monoaromatic hydrocarbons by using oxygen-releasing compounds in the PRBs [49,56,69].

The basic concept of biotic reduction, biotic oxidation, is to supply an electron donor along with nutrient materials to be used by microorganisms to break down the contaminants. Leaf mulch, wheat straw and sawdust can be used as electron donors, and municipal waste can be used as a nutrient material. Dissolved sulphate in the wastewater is a good electron acceptor, which can oxidate organic materials and can consume acidity coupling with metal reduction as shown in the below Equations (5) and (6):

(5) $$2CH_{2}O_{\left[Solid\;or\;oganic\right]}+SO_{4}^{2-}+H^{+}=2CO_{2}+2H_{2}O+HS^{-}$$

(6) $$Me^{2+}+HS^{-}=MeS_{\left[solid\right]}+H^{+}\;where\;Me=metal.$$

(4) Chemical Precipitation

This process consists of contaminants removal as hydroxides (Equation (7)) and carbonates (Equation (8)) via mineral precipitation resulting from increased pH. Firstly, contaminants are reduced to a less soluble species, and finally, they are retained as minerals in the barrier. Limestone (CaCo_3_) and apatite [Ca_5_(PO_4_)^3^(OH)] are commonly used in chemical precipitation

(7) $$Me^{2+}+2{\left(OH\right)}^{-}=Me{\left(OH\right)}_{2}$$

(8) $$Me^{2+}+HCO_{3}^{-}=MeCO_{3}+H^{+}$$

A summary of the available and common reactive media is presented in Table 3; the geochemical process, nature of contaminants, reactivity and availability are significant factors in the selection of the best convenient reactive media in remediating groundwater.

## 4. Modelling of Sorption Process

“Sorption” refers to the physical or chemical process in which a substance becomes in contact with another, which consists of two processes:

(1) “Adsorption” is a surface process; substances transfer from their aqueous phase (liquid or gas) to the solid phase surface that provides a surface for adsorption known as “adsorbent”; the species transformed from the aqueous phase to the surface of the solid phase is called “adsorbate” [62]. The existence of nitro groups on the adsorbate stimulating adsorption, hydroxyl, azo groups increases the adsorption rate, while the presence of sulfonic acid groups decreases adsorption [70].
(2) “Absorption” is defined as the whole transfer of substances from one phase to another without forces being applied to the molecules. The relationship governing the transfer of substances in aqueous porous media and the mobility of substances from liquid or gas states to the solid state is referred to as “isotherm” [71]. Adsorption isotherms is curvy relationships connecting the equilibrium concentration of a solute on the surface of an adsorbent (*q_e_*) to the concentration of solute in its aqueous state (*C_e_*); both phases should be in contact with each other [70,72].

### 4.1. Sorption Isotherm Models

Several isotherm models are used to describe sorption parameters and the adsorption of pollutants as follows:

#### 4.1.1. Freundlich Model

In 1909, Freundlich gave an imperial relationship that describes the capability of a unit mass of solid to adsorb gas in the presence of pressure. The Freundlich adsorption isotherm is a curve correlation between a solute concentration on a solid’s interface and the solute concentration in the adjacent aqueous environment [73]. The Freundlich isotherm model describes absorption in the terms of adsorbate concentration as follows:

(9) qe=KfCe1n

where $K_{f}\;\left(\frac{mg}{g}\right)\;$ is the coefficient of the Freundlich isotherm, *n* < 1, which describes the empirical coefficient expresses the amount of sorption [72,74,75]. $(K_{f)}\;and\;$(n) can be calculated by solving equation xx logarithmically and plotting $\ln q_{e}$ verses $\ln C_{e}$ where $K_{f}=10^{y-intercept}$ and the slop of ($\frac{1}{n}$) as shown below:

(10) $$\ln q_{e}=\ln K_{f}+\frac{1}{n}\ln C_{e}$$

According to the Freundlich isotherm, the sorbet contaminants is directly proportional to their concentration at a small amount and decreases when contaminants accumulate at the surface of the reactive media [76].

#### 4.1.2. Langmuir Model

The theoretical Langmuir isotherm model has been derived to describe the physical besides the chemical adsorption, as well as quantifying and describing the sorption on sites located on the adsorbent. Langmuir assumes the following [70,71,76]:

- Each adsorbate molecule is to be adsorbed on a well-defined binding site on the adsorbent, and adsorption reaches saturation when all these sites are occupied.
- Each active binding site on the adsorbent interacts with one adsorbate molecule only.
- No interaction existed between adsorbed molecules. All sites are homogeneous (energetically equivalents).
- The surface is uniform, and monolayer adsorption occurs.

Accordingly, the equation of the Langmuir isotherm model is:

(11) $$q_{e}=\frac{q_{m}bC_{e}}{1+C_{e}}$$

where $C_{e}$ (mg/L) represents the concentration of solute in the bulk solution at the equilibrium state. $q_{m}$ (mg/g) represents the maximum adsorption capacity. b is a constant that represents sorption free energy. $q_{e}$ (mg/g) represents the amount of the adsorbed solute by a unit weight of adsorbent within the equilibrium conditions. The Langmuir equation’s constant can be determined with the linearization of Equation (12) as follows:

(12) $$\frac{C_{e}}{q_{e}}=\frac{1}{q_{m}b}+\frac{1}{q_{m}}C_{e}$$

This equation describes that $\frac{C_{e}}{q_{e}}$ is plotted as a function of $C_{e}$, the parameters of $q_{m}$ and b  are determined from the slope ($\frac{1}{q_{m}}$) with y-intercept ($\frac{1}{q_{m}b}$) linear regression to Equation (12) [76].

#### 4.1.3. Temkin Model

The Temkin isotherm assumes that heats of adsorption would more often decrease than increase with the increase in solid surface coverage. It takes into account the adsorbing species–adsorbent interaction. Temkin isotherm has the following formula:

(13) $$q_{e}=\frac{RT}{b_{Te}}\ln(a_{Te}C_{e})$$

where R represents gas universal constants (8.314 J/mol K). T is the absolute temperature (K). $a_{Te}$ and $b_{Te}$ are constants.

#### 4.1.4. Brunauer–Emmett–Teller (BET) Model

The BET was developed based on the Langmuir model in an attempt to minimize the Langmuir isotherm restrictions. This isotherm assumes that more molecules can be adsorbed on the monolayer, and it is possible within this isotherm that bi-layer (multi-layer) adsorption will occur. This isotherm could be proclaimed as:

(14) qe=qmbCeCs−Ce1+b−1CeCS

where $q_{m}$ is the maximum adoption capacity, b represents a dimensionless constant, and $C_{s}$ is the concentration in the case of saturated sites and homogenous surfaces.

### 4.2. Kinetic Models

Adsorption kinetic models are important to describe the solution uptake rate and adsorption required time [74,75,77]; these models providing a description for the sorption process onto the sorbents. The sorption mechanism occurs in three steps; the first one is the diffusion of adsorbate through the aqueous phase surrounding the adsorbent; secondly, the diffusion of adsorbate in the pore of the particle (intrapore diffusion); finally, the adsorption occurrence due to physical or chemical interaction between the adsorbate and adsorbent [75,78,79]. However, three kinetic models are used to describe the sorption mechanism and the predominated stage as follows:

#### 4.2.1. Pseudo-First-Order Model

A model that is quantified according to Equation (15) below:

(15) $$\left(\frac{d_{qt}}{d_{t}}\right)=k_{1}\left(q_{e}-q_{t}\right)$$

where $q_{e}$ is the contaminant’s amount sorbet in equilibrium conditions (mg/g), $q_{t}$ represents a contaminant’s quantity sorbet during any given time (t) (mg/g), $k_{1}$ is a constant rate of pseudo-first-order adsorption (min^−1^).

The pseudo-first-order equation has been integrated at boundary conditions of *t* = 0 to *t* = *t* and $q_{t}$ = 0 to $q_{t}=q_{e}$, then transferred to a linear form as shown in Equations (16) and (17) [80].

(16) $$\mathrm{Linear}\;\mathrm{form}:\;\log\left(q_{e}-q_{t}\right)=\log q_{e}-\left(\frac{k_{1}}{2.303}\right)t$$

(17) $$\mathrm{Nonlinear}\;\mathrm{form}:\;q_{t}=q_{e}\;\left(1-e^{-k_{1}t}\right)$$

For this kinetic model, log$\left(q_{e}-q_{t}\right)$ must be plotted against time interval; if the intercept of $q_{e}$_theoretical_ differs than $q_{e}$_experimental_, then the reaction does not follow the model of the pseudo first order.

#### 4.2.2. Pseudo-Second-Order Model

The kinetic model of pseudo-second-order adsorption is applicable for small initial concentrations to calculate the initial sorption rate [74]. The pseudo-second-order equation for the sorption rate has the following form:

(18) $$\frac{t}{q_{t}}=\frac{t}{q_{e}}+\frac{1}{k_{2}q_{e}^{2}}$$

where *q_t_* is the magnitude of adsorbate, which is adsorbed by an adsorbent (mg g^−1^) at a given time (min), $q_{e}$ represents the amount of adsorbate adsorbed (mg g^−1^) in equilibrium conditions. $k_{2}$ is a constant of the second-order sorption rate (mg (mg min)^−1^) [80].

#### 4.2.3. Intra-Particle Diffusion Model

In 1962, Weber and Morris proposed the kinetic model of intra-particle diffusion, and it has been used for the analysis of adsorption kinetics of lead ions by adsorbent (CHAP) [76,80,81]. Based on this model, the uptake graph of ($q_{t}$) versus the squared root of time ($t^{0.5}$) must be linear in the overall adsorption process; in addition, if the line intersects with the origin, then the intra-particle diffusion is the predominant adsorption process. The *k_d_* represents the intra-particle diffusion initial rate (mg (mg min)^−1^), which could be calculated through the following formula:

(19) $$q_{t}=k_{d}t^{0.5}$$

where *q_t_* represents the amount of sorbate on the solid phase (surface of sorbent) at any time *t* (mg g^−1^), and t represents time (min).

## 5. Contaminant Transport Equation and Breakthrough Curves

**Figure 1:**
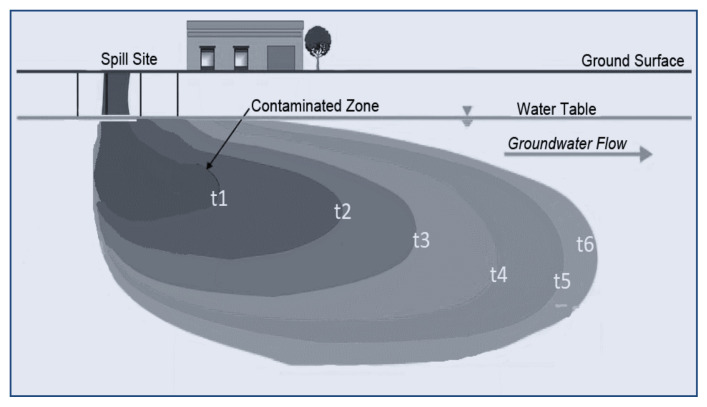
Contaminants concentration development in groundwater (“t1, t2–t6” are time intervals).

Soil is a dynamic system in which toxic contaminants are used as a sink or a pathway. When contamination occurs on the surface soil, some of these contaminants will percolate under the water table and form a plume of contaminants. This plume will be developed over time (*t*), and contaminants will be driven downstream, as shown in Figure 1. It is very important to understand how these contaminants will dissolve in the flow and how they will be carried out downstream; it is very important to discover the concentration of these contaminants as a function of time. The predominant mechanism for the attenuation and retardation of contaminants is sorption. Sorption phenomena will happen when the solid phase of the environment attenuate these contaminants, which will lead to contaminants being removed from the water, and the concentration of pollutants will be reduced downstream. The transport mechanism of pollutants in a saturated environment is the advection that carries contaminants without mixing. The hydrodynamic dispersion is driven by molecular diffusion and mechanical dispersion. If the hydraulic dispersion goes to zero, then the transport will be conservative, and there will be no retardation or any attenuation to the contaminants; on the contrary, if there is retardation to the contaminates, then the concentration of contaminants will be reduced at the downstream by the effect of sorption.

### 5.1. Modeling of Contaminants Transport

#### 5.1.1. Advection

In the advection, contaminants transport downstream along with the flow with advective velocity. It is the physical transport of contaminants across the space:

(20) $$V_{x}^{a}=\frac{V_{darcy}}{effective\;Porosity\;\left(n\right)}$$

where $V_{x}^{a}$ is the linear advective velocity.

(21) $$\mathrm{In}\;\mathrm{case}\;\mathrm{of}\;\mathrm{saturation}:\;V_{x}^{a}=\frac{V_{darcy}}{Volumatric\;moistuer\;containt\;\left(\theta \right)}$$

Darcy velocity is given by the meaning of Darcy law, which is:

(22) $$V_{darcy}=K.K_{r}.\theta .\frac{\partial_{h}}{\partial_{x}}$$

where (K) is the hydraulic conductivity, ($K_{r}$) is the relative conductivity, (θ) is the volumetric moisture content, and ($\frac{\partial_{h}}{\partial_{x}}$) is the head gradient in the x-direction.

(23) $$\mathrm{The}\;\mathrm{advective}\;\mathrm{flux}\;(F_{x})=V_{x}^{a}\;.\;n\;.c$$

where (F_x_) is the advective flux ($\frac{Kg}{t.m^{2}}$), ($V_{x}^{a}$) is the advective liner velocity ($\frac{m}{sec}$), (n) is the effective porosity, and (c) is the concentration of contaminants ($\frac{kg}{m^{3}}$)

(24) $$\mathrm{The}\;\mathrm{conservative}\;\mathrm{equation}\;\mathrm{is}\;n\frac{\partial c}{\partial t}=-\left(\frac{\partial F_{x}}{\partial x}+\frac{\partial F_{y}}{\partial y}+\frac{\partial F_{z}}{\partial z}\right)$$

Substitute $\;F_{x}$, $F_{y}\;$ and $F_{z}\;$ in the conservative equation:

(25) $$n\frac{\partial c}{\partial t}=-\left(V_{x}^{a}n\frac{\partial_{c}}{\partial_{x}}+V_{y}^{a}n\frac{\partial_{c}}{\partial_{y}}+V_{z}^{a}n\frac{\partial_{c}}{\partial_{z}}\right)$$

In the saturated medium, (n) = 1.

(26) $$\frac{\partial c}{\partial t}=-\left(V_{x}^{a}\frac{\partial_{c}}{\partial_{x}}+V_{y}^{a}\frac{\partial_{c}}{\partial_{y}}+V_{z}^{a}\frac{\partial_{c}}{\partial_{z}}\right)$$

#### 5.1.2. Hydrodynamic Dispersion

##### Molecular Diffusion

In a stagnant fluid, diffusion is the process of molecules random movement. It is basically driven by the concentration gradient and occurs by the Brownian motion. Therefore, diffusion usually increases with the increment of entropy.

In general, diffusion follows Fick’s first law:

(27) $$F=-D_{d}\frac{\partial_{c}}{\partial_{x}}$$

where (F) is the mass of solute per unit area per unit time ($\frac{M}{\frac{L^{2}}{T}}$), ($D_{d}$) is the diffusion coefficient ($\frac{L^{2}}{T}$) ≈ $10^{-9}$ ($\frac{m^{2}}{sec}$), and ($\frac{\partial_{c}}{\partial_{x}}$) is the concentration gradient ($\frac{\frac{M}{L^{3}}}{L}$).

According to the mass conservation of dissolved contaminants:

Accumulated contaminants = mass of contaminants in—the mass of contaminants out

The time-dependent concentration equation is:

(28) $$n\frac{\partial_{c}}{\partial_{t}}=-\left(\frac{\partial F_{x}}{\partial_{x}}+\frac{\partial F_{y}}{\partial_{y}}+\frac{\partial F_{z}}{\partial_{z}}\right)$$

n = 1 in a saturated medium.

Substituting Fick’s first law in Equation (28)

(29) $$\frac{\partial_{c}}{\partial_{t}}=D_{d}\left(\frac{\partial^{2}c}{\partial_{x^{2}}}+\frac{\partial^{2}y}{\partial_{y^{2}}}+\frac{\partial^{2}z}{\partial_{z^{2}}}\right)$$

For a one dimensional flow:

(30) $$\frac{\partial_{c}}{\partial_{t}}=D_{d}\frac{\partial^{2}c}{\partial_{x^{2}}}$$

**Figure 2:**
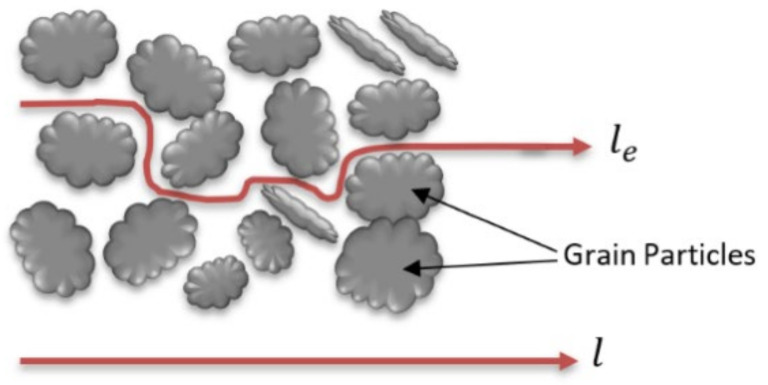
Determination of tortuosity in a porous medium.

The diffusion coefficient ($D_{d}$) here is the free diffusion coefficient (i.e., in water); if the flow medium is porous, then the effective diffusion coefficient ($D^{*}$) is used due to the effect of the tortuous flow path:

(31) $$D^{*}=w.D_{d}$$

w is related to the tortuosity (T): T = $\frac{l_{e}}{l}$ ≥ 1 as shown in the below Figure 2; laboratory studies showed that 0.01>w ≤0.5

##### Mechanical Dispersion

There is a number of mechanisms that lead to the assurance of the mechanical mixing of contaminants in the aquifer as follows:

(a) Mechanical dispersion due to pore size

**Figure 3:**
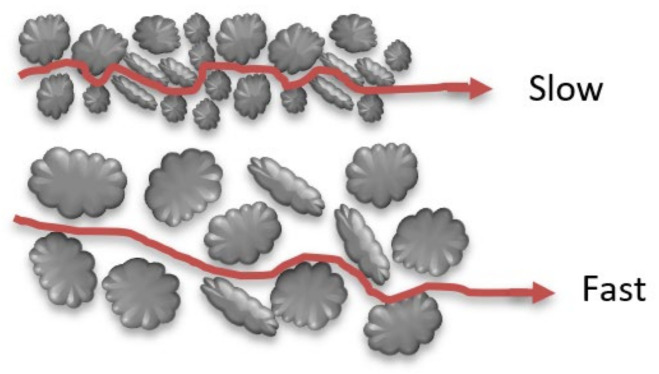
Mechanical dispersion due to pore size.

When dissolved contaminants pass through a porous medium, pore size will affect the hydraulic conductivity of this media; when particles are fine, porosity will be below, and the advective velocity will be slow, as shown in Figure 3.

(b) Mechanical dispersion due to path length

**Figure 4:**
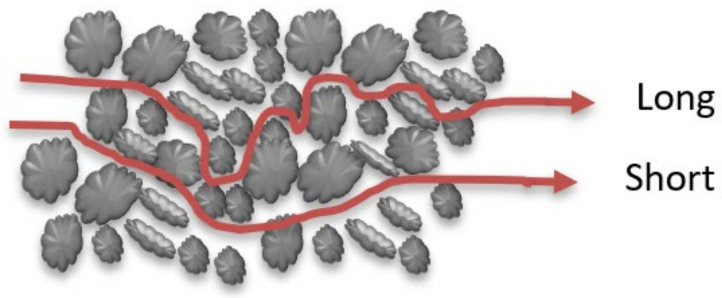
Mechanical dispersion due to path length.

If a pore is medium, the mechanical mixing may happen due to the effect of the length of the pathway, which will be passed by the dissolved contaminants. Each molecule of contaminants will pass through a different pathway that is unequal with the pathway of other particles, as illustrated in the below Figure 4.

(c) Tylor dispersion

**Figure 5:**
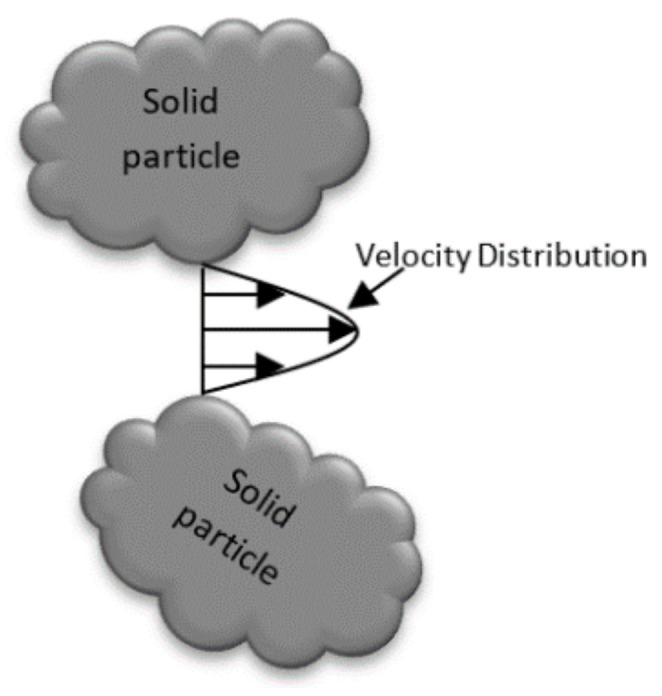
Tylor mechanical dispersion.

Taylor mechanical dispersion occurs when dissolved contaminants pass around the aquifer’s solid particles. Solids pass faster in a middle way between two particles than another pass near a solid particle, as shown in Figure 5. This is because the linear velocity in the centre of pores is greater than that near the edge of solid particles.

All the above mechanisms lead to mechanical mixing for solute contaminants in both the longitudinal direction (with the main flow direction) and the transverse direction (out of the main flow direction).

The coefficient of mechanical dispersion (*D*) is related to aquifers’ dispersivity (α), which reflects the extent to which the aquifer is dispersive and the advective velocity of flow.

(32) $$D_{L}=\alpha_{L\;}.\;V_{L}^{a}$$

(33) $$D_{T}=\alpha_{T}.\;V_{T}^{a}$$

where ($D_{L}$) and ($D_{T}$) are the mechanical dispersion coefficient in the longitudinal and transverse directions (m^2^/sec), respectively. ($\alpha_{L\;}$) and ($\alpha_{T}$) are the longitudinal and transverse dispersivity (m), respectively. ($V_{L}^{a}$) and ($V_{T}^{a}$) are the longitudinal and transverse advective velocity (m/sec).

Hydrodynamic dispersion = molecular diffusion + mechanical dispersion

(34) $$\frac{\partial_{c}}{\partial_{t}}=D_{L}\frac{\partial^{2}c}{\partial_{x^{2}}}+D_{T}\frac{\partial^{2}c}{\partial_{y^{2}}}-V^{*}\frac{\partial_{c}}{\partial_{x}}$$

(35) $$\mathrm{Where}:\;D_{L}=\alpha_{L\;}.\;V_{L}^{a}+D^{*}$$

(36) $$D_{T}=\alpha_{T\;}.\;V_{L}^{a}+D^{*}$$

In the low permeability medium, the permeability is near to zero; in this case, there will be no effect on the mechanical dispersion, and only the diffusion will be predominant.

#### 5.1.3. Advection–Diffusion Equation

The theory of contaminants transport model in porous media is subjected to a partial differential equation governing space and time. The theory incorporates four different processes, all merged in one equation; one process is advection, which means that a substance follows the direction of water (driven by water flow) and itself moves with convection. The second process is dispersion, which is caused by the heterogeneity of pollutants, and a package of contaminants will move faster than the others. Then, there is a chemical reaction, which described by a kinetic equation. Finally, there is the adsorption to the soil, which means that the contaminant may spend some of its time tied to the solid phase and sometimes in the mobile water. The equation that describes all of this is the advection–dispersion equation, as follows:

(37) $$\frac{\partial m}{\partial t}=\frac{\partial \left(nC\right)}{\partial t}=-\left(\frac{\partial F}{\partial x}+\frac{\partial F}{\partial y}+\frac{\partial F}{\partial z}\right)\mp r$$

In the above equation, the change in mass per unit volume (*m*) of the contaminants due to the reactions within the aquifer is referred to as (r).

(38) $$F_{x}=(V_{x}nC)-(nD_{x}\frac{\partial C}{\partial x})\;$$

where ($F_{x}$) is the total flux in the (X) direction. (*V_x_ n C*) is the addictive flux, and (– (*n D_x_*$\frac{\partial C}{\partial x}$)) is the dispersive flux.

Substituting ($F_{x}$) in Equation (37) for (x, y and z) directions:

(39) $$\frac{\partial \left(nC\right)}{\partial t}=-\left[\frac{\partial }{\partial x}\left(V_{x}nC-nD_{x}\frac{\partial C}{\partial x}\right)\right]-\left[\frac{\partial }{\partial y}\left(V_{y}nC-nD_{y}\frac{\partial C}{\partial y}\right)\right]-\left[\frac{\partial }{\partial z}\left(V_{z}nC-\;nD_{z}\frac{\partial C}{\partial z}\right)\right]\pm r\;$$

In the 1D flow, with a constant dispersion coefficient and constants porosity in space and time (=1 in a saturated medium), the equation of advection–dispersion can be written as:

(40) $$\frac{\partial C}{\partial t}=D_{x}\frac{\partial^{2}C}{\partial x^{2}}-V_{x}\frac{\partial C}{\partial x}\pm \frac{r}{n}$$

The term (*r*) is considered an important factor in the attenuation of contaminants in a porous media, which is related to the sorption, the predominant process of contaminants attenuation in a permeable reactive barrier during contaminants’ mass transfer. Generally, (*r*) depends on the bulk density (*ρ_b_*) of the medium and the amount of contaminants sorbed (q) with time, thus:

(41) $$r=\rho_{b}\frac{\partial q}{\partial t}$$

By substituting the value of (r) (Equation (41)) in Equation (40), the advection–dispersion will be as follows:

(42) $$\frac{\partial C}{\partial t}=D_{x}\frac{\partial^{2}C}{\partial x^{2}}-V_{x}\frac{\partial C}{\partial x}-\frac{\rho_{b}}{n}\frac{\partial \left(q\right)}{\partial t}$$

The sorption process is represented in the above equation by the term $\frac{\rho_{b}}{n}\frac{\partial \left(q\right)}{\partial t}$, (*q*) represents contaminants concentration that sorbed on the solid phase of the reactive media, which can be described by the Langmuir or Freundlich isotherm models as a function of concentration. Equation (42) can be rewritten as follows:

(43) $$R\frac{\partial C}{\partial t}=D_{x}\frac{\partial^{2}C}{\partial x^{2}}-V_{x}\frac{\partial C}{\partial x}$$

where (R) is the retardation factor, which reflects the effect of retardation of contaminants during its transport to the downstream.

The “breakthrough curve” describes the relationship between the concentration of contaminant vs. time, which is an important tool for design and optimizes the sorption in a field-scale PRB by relating the data obtained from laboratory columns to the field scale breakthrough curves. In a continuous constant influent of contaminants, the breakthrough curve will be shaped as (S); the best point on this curve is referred to as the breakthrough point, which has an outlet concentration of contaminants that matches the desired concentration in water. A summary of empirical and theoretical models used to predict the breakthrough curves are described below:

- Bohart–Adams model

The purpose of performing column experiments is to calculate the relationship between the concentration and time, the breakthrough curve in addition to calculate the maximum adsorbent capacity of adsorption. Results will be used to design a full-scale adsorption column. The Bohart–Adams model is one of the models that has been formulated to fulfil this purpose; it has been based on the rate of surface reaction theory [82]. This model has been built on the following assumptions [48]:

1. This model can describe the concentration at low levels ($C«C_{0}$) (C=0.15 $C_{0}$).
2. When $t\to \infty ;q_{0}\to N_{0\;}$ with saturation concentration.
3. The external mass transfer is limiting adsorption speed.
4. The Bohart–Adams model has the following formula:
  (44) $$\frac{C}{C_{0}}=\frac{1}{1+\exp\left(KN_{0}\frac{Z}{U}-KC_{0}t\right)}$$
  where $C_{0\;}$ and C represents the instantaneous (initial) concentration of the pollutant in solution (mg/L). K is the kinetic constant (L/g/min). $N_{0}$ represents the congestion concentration (mg/L). Z represents column bed depth (cm). t represents the time of service (min), and U is the velocity of the flow (cm/min).

- Thomas model.

The Thomas model is widely used to calculate adsorbent maximum adsorption capacity. It uses data obtained from continuous column experiments. The Thomas adsorption column is given below:

(45) $$\frac{C}{C_{0}}=\frac{1}{1+\exp\left[\frac{K}{Q\;}qM-K_{T}C_{0}t\right]}$$

where $C_{0\;}$ and C are the concentrations of influent (mg/L). $K_{T}$ is the constant rate (mL/mg/min), q represents the higher adsorption capacity (mg/g), M represents an adsorbent quantity in the column (g), t is the time of adsorption (min), and Q is the feed flow rate (mL/min). The Thomas model is based on the following assumption:

1. No dispersion is driven.
2. The Langmuir isotherm coincide with the equilibrium state.
3. Adsorption kinetics (K) should follow the rate of pseudo-second-order law.

- Yoon–Nelson model

In this model, the decreasing probability of each adsorbate is proportional to its breakthrough adsorption on the adsorbent. The following formula is a representation of this model:

(46) lnCCF−C=KYNt−t12kYN

where $K_{YN}$ represents the Yoon–Nelson rate constant. The Yoon–Nelson model is limited by its rough form.

- Clark Model

Clark’s breakthrough curves were based on the mass transfer principle in conjunction with the Freundlich isotherm. Clark has developed his breakthrough curves as follows:

(47) $${\left(\frac{C}{C_{0}}\right)}^{n-1}=\frac{1}{1+A.e^{-rt}}$$

where n represents the exponent of the Freundlich isotherm, A and r represents the parameters of the kinetic equation.

- Wang model

Wang et al. (2003) invented a new model based on the mass transfer model. It has been used as a solution of Co and Zn ions in a fixed bed under the following assumptions:

1. The adsorption mechanism is isothermal.
2. The mass transfer equation is as the following:
  (48) $$-\frac{d_{y}}{d_{t}}=K_{w}xy$$
  where *K_w_* represents the kinetic constant, the fraction of adsorbed metal ions is represented by y. (with x+y=1),  x represents the fraction of metal ions moving through the fixed bed.

1. There is symmetry in the breakthrough curve.
2. The axial dispersion in the column is negligible.

By integrating the above equation and presuming that $y=y_{w}$ at $t=t_{w}$. w=0.5, the entire breakthrough equation can be expressed as:

(49) t=t12−1Kwln11−x

where (x) can be expressed as:

(50) $$x=\frac{C}{C_{F}}$$

Finally, the Wand model, similar to the Yoon–Nelson model, cannot provide enough detail on the adsorption mechanism.

## 6. Review of Previous Research on the Use of PRBS

The first permeable reactive barrier was constructed at a Canadian air force base in (1991) [83]; since that date, many studies have been conducted to examine the PRB’s efficiency. There were 624 publications that discussed the permeable reactive barrier from 1999 to 2009 [84,85]. Previous research has been conducted to study the ability of different reactants to remediate different pollutants in the permeable reactive barrier. The following is a list of the most important scientific studies.

The remediation of groundwater contaminated by chlorinated ethenes such as vinyl chloride (VC), dichloroethene (DCE) and trichloroethene (TCE) was studied using in situ biodegradation with a special functional microorganism known as *Burkholderia cepacia* ENV435 [86]. The researchers chose these microorganisms for many important characteristics, such as their good adhesion ability to aquifers’ solids; in addition, these microorganisms can establish an organized existence without the need to induce co-substrates. Furthermore, these organisms can grow in a high density in fermenters (−100 g/L), and finally, they can accumulate high internal energy, which this microorganism can use to resist the effect of chlorinated solvents and survive. Results showed the concentrations of VC, DCE and TCE decreased by 78% after two days of organism injection.

The output of a pilot-scale PRB for the remediation of chlorinated volatile organic compound-contaminated groundwater (VOCs) has been investigated. This study used a granular zero-valent iron reactive barrier, which was mounted in a funnel with a gate mechanism. Results showed that consistent VOC degradation was observed over the research period. It is observed that the degradation mechanism is due to pH increment, which leads bicarbonate ($HCO_{3}^{-}$) to convert to carbonate ($CO_{3}^{2-}$), the carbonate combines cations ($Ca^{2+},\;Fe^{2+},\;Mg^{2+},\;\mathrm{etc}.$) in solution, which form mineral precipitates. It is observed that mineral precipitates formed in the reactive media represented as an unconquerable limitation to the treatment process [87].

A zero-valent iron PRB’s effectiveness in eliminating chlorinated aliphatic hydrocarbons (CAHs) has been investigated. The contact of reactive media (ZVI) with the CAHs in an aqueous environment caused a rise in the pH; this resulted in the precipitation of carbonate minerals and a loss of 0.35% of the porosity in the reactive fraction of the PRB [88].

The rapid evolution of the PRB’s application from a full in situ implementation on a laboratory level to treat groundwater polluted by various types of inorganic and metals was assessed [89]. This study concluded that different reactive media can be used in the preamble reactive barrier to remove inorganic compounds, such as the use of zero-valent iron PRB to remove TC, U and Cr from groundwater. Furthermore, solid-state organic carbon may be used to extract dissolved solids associated with acid-mine drainage. According to this research, there are different mechanisms for the treatment of inorganic anions; for example, the rate of Cr(VI), TC (VII), U(VI) and NO_3_ could be successfully decreased by the mean of reduction using zero-valent iron (Fe^0^). According to a monitoring program for a Cr(VI)-contaminated area, the concentration of Cr(VI) has decreased from 8 mg L^−1^ to > 0.01 mg L^−1^, owing to a decrease in Eh and an increase in pH.

At a former uranium production site in Monticello, Utah [90] investigated the design and efficiency of a PRB in extracting arsenic, uranium, selenium, vanadium, molybdenum and nitrate. In this study, field and laboratory column tests have been performed. The reactive media in PRB was the zero-valent iron. After one year from PRB installation, the performance of ZVI–PRB is described by the reduction in concentrations of elements up-gradient and down-gradient of the barrier. The inlet concentrations of arsenic, manganese, molybdenum, nitrate, selenium, uranium and vanadium were 10.3, 308, 62.8, 60.72, 18.2, 396 and 395 µg/L, respectively. These concentrations have reduced to be >0.2, 117, 17.5, >65.1, 0.1, >0.24 and 1.2 µg/L, respectively. The removal mechanism for these radionuclides is by reducing uranium to lower molecules along with precipitation. Additionally, adsorption is another chemical process that leads to a reduction in these elements.

The use of a reactive biological barrier to remove nitrate pollutants has been investigated. The autotrophic sulphur-oxidizing bacteria has been used as an electron donor, and sulphur granules have been used as a biological agent. Sulphur-oxidizing bacteria colonized the sulphur particles and removed nitrate, according to the findings. The best operation conditions have been investigated, and it was found that an environment near the neutral pH achieved 90% removal of nitrates [91].

The efficacy of a ZVI barrier mounted in the field in eliminating chromium solid-phase association has been studied, and the removal efficiency after 8 years of operation has been investigated. Results showed that ZVI has the ability to reduce the concentration of Cr from an average <1500 µg/L to about >1 µg/L. The reduction in Cr(VI) to Cr(III) along with the oxidation of Fe(0) to Fe(II) and Fe(III), resulting in Fe(III)-Cr(III) precipitating as oxyhydroxides and hydroxides, has been discovered to be the most common Cr removal mechanism. It was also discovered that the reacted iron produced a coating of goethite (α-FeOOH) with Cr, resulting in precipitation [92].

Experiments have been performed to discover the efficiency of seven selected organic substrates in removing inorganic nitrogen in the form of NO_3_^−^, NO_2_^−^ and/or NH_4_^+^ in a denitrification PRB in batch scale experiments. Softwood, hardwood, coniferous, mulch, willow, compost and leaves were all reactive materials. The softwood was found to be suitable for use as a reactive medium in PRB due to its very good ability to denitrify nitrogen. Reduction in nitrate was due to the effect of denitrification (which represents 90% of the nitrate removal of which the dissimilatory nitrate reduction to ammonia (DNRA) represents 10% of the removal process [93].

The efficacy of activated carbon PRB for removing cadmium from contaminated groundwater has been investigated. The original cadmium concentration was 0.020 mg/L, but after it passed through a PRB of activated carbon, the polluted plume was adsorbed, and the cadmium concentration was nearly zero for the first three months. After that, the barrier became saturated, but the effluent cadmium concentration remained below the quality limit of 0.005 mg/L for more than seven months [94].

The use of polyvinylpyrrolidone (PVP-K30)-modified nanoscale ZVI in removing tetracycline from liquid has been investigated. Tests revealed that PVP-nZVI consists of Fe(0) in the core and ferric oxides on the shell. PVP-nZVI will adsorb tetracycline and its degradation products, according to the findings. It is also observed that the adsorption of tetracycline has been reduced with time due to the formation of H_2_PO_4_^−^, which has a strong tendency to react with the mineral surface [95].

Tetracycline adsorption using graphene oxide (GO) as a reactive media has been investigated. Results showed that tetracycline formed a π–π interaction and cation–π bonds with the surface of GO, with the Langmuir and Temkin models providing the best fit isotherms for adsorption and the Langmuir model calculating a maximum adsorption capacity of 313 mg g^−1^. The kinetics of the adsorption model are also equipped with a pseudo-second-order model with a better sorption constant (k), 0.065 g mg^−1^ h^−1^ than other adsorbents, according to the results [96].

The design, construction and testing of a permeable barrier at the Casey station in Antarctica to remediate and avoid the spread of an old diesel fuel spill have been discovered. Five segments of a bio-reactive barrier were allocated and installed in the funnel and gate configuration, each segment divided into three zones; the first one is a slow-release fertilizer zone to enhance the biodegradation, the second zone is responsible for hydrocarbon and nutrient capture and degradation, while the third zone is responsible for cation capture and access to nutrients produced by the first zone. The first zone’s reactive media was a nutrient source, followed by hydrocarbon sorption materials (granular activated carbon plus zeolite); to extract cations nutrient released and accessed from the first region, sodium activated clinoptilolite zeolite is used. Oxygen delivery to the system was applied to enhance the microbial reactions. The function of each zone is the first zone to provide nutrients such as phosphorate to the microorganism. Due to its high surface area and microporous surface (500–1500) m^2^/kg, granulated activated carbon can adsorb hydrocarbon pollutants in the second zone. In the third zone, the Australian sodium zeolite is placed to capture any accessed ammonium cation from the solution due to its high ability to exchange ions with ammonium. Tests and results showed that the ion exchange of zeolite best-controlled nutrient concentration, while the sodium zeolite captured any migrated ammonia from the groundwater. Additionally, results showed that the fuel is degraded in the PRB faster than in the hydrocarbon spill area field. In the cold world, activated carbon–PRB is a strong technology for removing hydrocarbons.

In batch and fixed-bed column experiments, the adsorption of tetracycline (TC) and chloramphenicol (CAP) was investigated by [97] using bamboo charcoal (BC) as a reactive medium. The predominant mechanism of TC and CAP adsorption on BC is π–π  electron-donor–acceptor (EDA), cation–π bond in combination with H-bond interaction, while the hydrophobic and electrostatic interaction has a minor effect on the adsorption. Results showed that BC has a strong adsorption capacity to TC and CAP; with increasing influent concentration and flow rate, adsorption efficiency improves. Surface diffusion was the most common mass transfer mechanism for antibiotic adsorption [98,99].

An overview of the use of PRBs in the remediation of a broad range of pollutants, demonstrating that it is a viable alternative to the pump-and-treat process, has been discussed by [85]. The most popular PRB reactive media, according to this study, is zero-valent iron (ZVI). Efficient PRB architecture requires accurate site characterization, groundwater flow and flow conditions requirements and ground flow modelling.

The potential efficiency of a microscale zero-valent iron PRB in removing tetracycline (TC) and oxytetracycline (OTC) with the formation of transformation products during the remediation have been discovered. To investigate the effect of solution pH, a series of batch experiments were carried out, including iron dose and environment temperature. Results showed that pH has a key factor controlling the efficiency of removal; increasing iron dose and working temperature also increased the removal efficiency. Pseudo-second-order model and Langmuir isotherm were found to be most fitted to adsorption kinetics and removal isotherms [100].

The effectiveness of removing copper ions Cu(II) and zinc ions Zn (II) heavy metals from groundwater using cement kiln dust and a sand PRB was investigated by [48]. In this research, the re-use of a very fine by-product powder resulted from the cement industry known as cement kiln dust (CKD) has been investigated to remove appointed heavy metals instead of throwing this CKD into the environment. The optimum weight ratio of CKD/sand, which provides the best remediation, has been investigated in column tests from 99 days of operation time. The remediation mechanisms were the adsorption/desorption, precipitation/dissolution and adsorption/desorption of the pollutants. Contaminant transport in porous media, as well as breakthrough curves, are also explored. Breakthrough curves refer to the relationship between the concentration of the contaminants at any time in any position in the domain. Results showed that the best CKD/sand ratio was (10:90 and 20:80) because other ratios showed a loss in the hydraulic conductivity and loss in groundwater flow due to the accumulation of contaminants mass in the voids between the sand causing clogging and flow loss.

The mechanism of remediating pharmaceutical pollutants (tetracycline) from groundwater using zero-valent iron coupled with microorganisms as reactive media has been investigated by [55]. In this research, three PRB columns have been studied, beginning with columns filled by zero-valent iron, the second with zero-valent iron and microorganisms and, finally, the third one with microorganisms. Results revealed that zero-valent iron has the best effect on removing tetracycline. Removal efficiency reaches 50% while it was 40% with zero-valent iron and microorganisms’ PRB and 10% by the effect of microorganisms’ PRB. The mechanism of this reaction is that the zero-valent iron (Fe^0^) has been adsorbed and reduced tetracycline, Fe^0^ converted to Fe^+2^ and Fe^+3^, and the tetracycline has been degraded.

The use of a bio-PRB coupled with a good aeration system to remediate groundwater polluted with nitrobenzene and aniline have been studied. To degrade the NB and AN, suspension-free cells of the degrading consortium and the immobilized consortium were used in this study. Results showed that both AN and NB were completely degraded within 3 days in the immobilized consortium, while it needs 3–5 days to degrade using the free cells. It was also discovered that in the presence of oxygen, the removal efficiency of NB and AN was increased [56].

In a permeable reactive barrier, [101] investigated the effect of MnO_2_ and its mechanism of tetracycline elimination. The zero-valent iron serves as the reactive media in this PRB. In this research, three PRB columns were studied, the first one with ZVI, the second had ZVI-MnO_2_, while the third consisted of MnO_2_ only. Results show that the ZVI in the presence of MnO_2_ is the most effective material in removing TC. Its removal efficiency reached 85%, while the ZVI removed about 65% and the MnO_2_ removed 50% of TC. This research revealed that MnO_2_ accelerated the transformation of Fe^2+^ to Fe^3+^, then the Fe^3+^ degraded tetracycline. The functional group that played the predominant role in this reaction is the hydroxyl radical produced in this process.

A series of laboratory and field studies in the Ukrainian city of Zhovty Vody has been performed to assess the reliability of a reactive barrier made up of zero-valent iron and organic carbon mixtures to remediate uranium-contaminated groundwater. In these studies conducted by [102], three reactive media were examined. The first was zero-valent iron, which was used to study the sorption, reduction and precipitation of redox oxyanions; the second was the phosphorate materials, which has been used to transfer the dissolved materials to other phases; the third was bioremediation materials and organic carbon substrates. The study revealed that the treatment mechanism of the uranium is sorption by the ZV, and it also observed that the microbes have the ability to sorb the uranium U(VI) to the bacterial cell walls. Due to the effect of enzymatic production, dissolved oxygen reduced first, then due to the effect of denitrification, UO_2_CO_3_ reduced to uranite and sulphate reduced to sulphide; finally, amorphous uranium oxide will be formed on the microorganism surfaces. In this research, new placement of the reactive media has been used in which rows of cylinders with iron reactive media have been placed instead of the regular funnel and gate placement; this placement reduced the in situ installation cost.

The effectivity of PRB made from sodium alginate/graphene oxide hydrogel beds (GSA) for the remediation of ciprofloxacin (CPX) antibiotic contaminating the groundwater has been investigated. In this research, the key factors affecting the performance have been studied, and longevity and the cost of PRB have been discussed, and a proper design for the PRB has been proposed. Results show that the adsorption capacity of CPX on the GSA was 100 mg for each gram of GSA at pH 7.0; the leading mechanism in the adsorption process was the pore filling, H-bonding, ion exchange, electrostatic interaction and hydrophobic interaction. The results indicate that the GSA’s ability to remove CPX from groundwater when used in a PRB is concrete evidence that GSA is a good option for removing CPX from groundwater [103].

The removal of tetracycline from aqueous solutions using binary nickel/nano zero-valent iron (NiFe) reactive media in column reactors has been studied. Results show that if a mixture of 20 mg/L of TC plus 120 mg/L of NiFe in a 90 min time of interaction, TC will be removed by 99.43%. In this research, sand particles loaded with reactive media (NiFe) have been used. Electrostatic interaction has been used to load the reactive media on sand particles. A Tc removal mechanism was investigated using UV-Visible spectroscopy, TOC, FTIR and SEM analysis [104].

The use of the PRB system in preventing the migration of radiocesium into groundwater using natural zeolite and sepiolite has been investigated. These reactive media are natural, low-cost materials. Two-dimensional bench-scale prototypes at the steady flow conditions have been used in the experiment. Information on the transport behaviour of radiocesium and changes in hydraulic conductivity were investigated in this study. It has been determined that the remediation phase would reduce hydraulic conductivity over time. As a result, by combining sand with reactive media, the PRB has been modified to achieve steady-state operating conditions of flow [105].

The effectivity of the use of PRB of cement kiln dust as a reactive media in an acidic environment (pH 3) to remediate groundwater contaminated with dissolved benzene has been studied by [9]. Experiments were performed for 60 days with batch and column tests. Results showed that benzine removing efficiency reached more than 90%, and the best CKD/sand ratio was 5/95, 10/90 and 15/85, which achieved the best hydraulic conductivity. Results also show that barrier longevity reached (half a year) when CKD was about 15%. FTIR test results showed that adsorption happened due to the formation of H bonding and cation.

The removal of meropenem antibiotic with a cement kiln dust (CKD) PRB through batch and continuous column experiments have been studied by [106]. Results showed that pH 7.0 had a 60 mg adsorption potential for every 1 g of CKD, according to the findings. Initial concentration, flow rate and influence have all had an impact on CKD efficiency. Meropenem adsorption occurred due to the O-containing functional group’s effect on the surface of CKD, which leads to an H-bonding and π–π and n–π EDA interaction (donor–acceptor) between the CKD and the meropenem, which all lead to the adsorption.

The sustained treatment of a bio-wall and its effectivity in remediating groundwater contaminated by chlorinated volatile organic compounds (TCE) after 10 years of bio-wall installation has been studied by [107]. The reactive medium used in this barrier was mulch, utilizing the benefit of its high cellulose content (<79%). This research investigates a reactive barrier of mulch (1615 m long × 10.7 m depth × 0.6 m thickness). This bio-barrier consisted of 42% mulch, 11% cotton, 32% sand and 15% rock to increase the permeability. It is estimated that groundwater retention time within the barrier is 2–50 days, while groundwater speed was (0.002–0.3 m/day). Contaminants were trichloroethene (TCE), tetrachloroethene (PCE), dichloroethene (DCE) and vinyl chloride (VC). After 10 years of the bio-wall installation, results showed that mulch bio-wall effectively degrades TCE from groundwater to daughter products, TCE concentrations remained below the USEPA maximum levels, while it was over these levels in the up-gradient side of the bio-wall. The microbial population, geochemical environment of the barrier was still active. Investigating the concentration patterns, microbial community and the geochemical environment of the bio-wall demonstrates that the bio-wall is an effective reductive to the volatile organic contaminants.

The effectiveness of a horizontal PRB with a reactive media of zero-valent iron to prevent the scattering of chlorinated solvent vapour in the unsaturated region was investigated by [108]. In this research, the potential feasibility of using PRBs placed in a horizontal direction was investigated. The reactive medium in this study was the zero-valent iron (ZVI) powder mixed with sand, and the TCE was tested as a model for the (VOCs). Tests were performed in batch reactors. Results showed after 3 weeks of treatment and based on the type of ZVI powder, the concentration of TCE vapour was reduced in a range of 35–99%. The ZVI’s best output is determined by the particular surface area.

The use of sewage sludge and cement kiln dust to produce hydroxyapatite nanoparticles has been investigated. The removal of tetracycline using the new formed hydroxyapatite were examined and the best operation conditions were 2 h contact time, dosage 0.4 g/50 mL, agitation speed 200 rpm with a mixture molar ratio Ca/P = 1.662, the removal efficiency reached 90% with a TC maximum adsorption capacity of 43.534 mg for each gram of hydroxyapatite filter cake. Results show that adding 10% sand (to enhance the hydraulic conductivity of the PRB) to the hydroxyapatite reduced the adsorption capacity to be 41.510 mg/g. XRD, FTIR and SEM analytical tests proved that the predominant mechanism for the remediation of TC is due to the adaptation on the hydroxyapatite surface. During the process, two functional groups, (-OPO3H-) and (CaOH2+), were formed, both of which are positively charged with the ammonium functional group and negatively charged with the phenolic diketone moiety of TC species. The removal of TC was also aided by the effect of hydrogen bonding and surface complexes formed between TC and Ca [109].

## 7. Conclusions and Perspective

In recent decades, there has been an increment in the dependence on groundwater as a major source of freshwater for daily human needs, but in many places, groundwater is being polluted by organic and inorganic contaminants. It is very important to remediate groundwater before use to prevent the spread of contaminants to the neighbour environment. Many techniques and reactive materials have been used in the remediation of contaminated groundwater; one of the most popular technologies is PRBs, which is considered an affordable technology. It allows the treatment of multiple pollutants if a multi-barrier is being used. In PRB technology, there is no adverse contamination that may happen, as contaminants will not be brought out to the surface. On the other hand, this technology may have some limitations, such as the difficulty of detailed site characterization required prior to the design of PRB, and only contaminants passing the PRB could be treated in addition to the limited field data for the longevity of the PRB, so the prospective tendency is to use new by-product materials to improve PRB performance. In this way, the environment will be saved by the disposal of these unwanted by-products and will be considered a (green) refreshment to the environment.

Groundwater contamination is now a global issue; solving this problem involves close coordination between scientists at universities and government agencies, as well as the industry and decision makers at all levels. The way ahead for solving this problem must include addressing the levels of groundwater contamination in different countries by using developed measures, techniques and policies. In addition, the variation of the influence of groundwater contamination in different countries must be well studied, including the effect on climatic regions and geological features. To study groundwater contamination in the future, groundwater scientists will need to adopt and apply new technologies such as artificial intelligence, “big data” analysis, drone surveys and molecular and stable isotope analysis technologies. Finally, governments, especially those with developing economies, need to invest more in groundwater and encourage researchers, training and research in this important, valuable field.

## Figures and Tables

**Table 1:** Inorganic pollutants presented in groundwater.

Contaminant	Source for the Groundwater	Problems	MCL (mg/L) (USEPA, 2018)	Reference
Aluminium	Groundwater passes through some kind of rocks. Mines discharge.	If present in drinking water, it could cause turbidity increment besides water discolouring.	0.05–0.2	[23]
Antimony	Municipal waste disposal. Industrial production and flame retardants such as glass manufacturing, ceramics and lead industry. Fireworks or explosives.	Cause a change in cholesterol and glucose concentrations in blood in laboratory animals exposed to risky levels of antimony during their existence. Decreases longevity. Has a biochemical changes in laboratory animals and toxic effect on neurobehavioral.	0.006	[23,24]
Arsenic	Industrial activities such as smelting of metals (zinc, lead, copper ore) Using pesticides. Naturally found in aquifers.	Liver, kidney and skin damage. Decrease blood haemoglobin. Chronic and acute toxicity. Can cause various forms of cancers. Hindrance of children’s development.	0.010	[25,26,27]
Barium	Naturally takes place in some kind of soils such as limestones and sandstones. Landfill leachate. Fertilizers and pesticides.	Cardiovascular and kidney diseases. Mental disorders. Metabolic syndrome.	2	[28,29]
Cadmium	Industrial and mining waste. Phosphate fertilizers. Landfill leachate.	High blood pressure. Replace zinc biochemically in the human body. Liver damage Destroy testicular tissues and blood cells (red).	0.005	[30]
Chloride	Industrial and domestic waste. Saltwater intrusion.	Changes in drinking water taste. At high levels, it can deteriorate water heaters, municipal pipes, pumps and works equipment.	250	[23,31]
Dissolved solids	Naturally found. Human activities such as landfill leachate, feedlots.	When presented, the water became unacceptable and objectionable to many. Affect the performance and life of water heaters.	500	[32]
Iron	Mining corroded metal, industrial waste. Naturally found in sediments and rocks.	Changing water taste. Affect plumbing fixtures and clothes colours in laundries.	0.3	[26]
Lead	Industry, mining, gasoline and plumbing.	Affect babies’ mental growth and can change red blood cells chemistry. Increase blood pressure. Probable carcinogen.	0.015	[28]
Zinc	Industrial waste, metal plating, is the major ion in sludge. Naturally, it is found in mining areas.	Cause a change to the drinking water taste. Toxic to plants if exposed to high levels.	5	[27]

**Table 2:** Organic contaminants, source to groundwater and their effects.

Contaminant	Source for the Groundwater	Problems	Reference
Volatile organic compounds (VOCs)	Anthropogenic activities. The pharmaceutical industry, dyes, polishes, inks, paints, disinfectants and spot removals industry Crude oil industry.	Can cause damage and cancer in the liver, skin irritation, weight loss, nervous system damaging and problems to the respiratory system.	[33,34]
Pesticides	The use of herbicides, insecticides, fungicides, rodenticides and algicides.	It causes headaches, poisoning, cancer.Problems to the nervous system and gastrointestinal disturbance.	[35]
Plasticizers, chlorinated solvents and dioxin	Solvents, pesticides, components of gasoline wood preservations.	Can cause cancer, problems in the nervous system, damage to the stomach and liver.	[29]
Pharmaceutical, antibiotics pollutants	Confined animal feed operation facilities and feedlots. The use of wastewater for groundwater recharge. Municipal solid waste landfills. Medical industrial activities.	The wide spread of antibiotics to the human and veterinary system caused a constant input of chemicals to the lifecycle, which caused the appearance of multi-drug-resistant bacteria.	[36]

**Table 3:** Reactive media for the remediation of groundwater contaminated by metals and radionuclides (Bronstein, 2005).

Type of Reactive Media	Predominant Remediation Approach
Activated carbon products	Remediation by adsorption
Products made of amorphous ferric oxyhydroxides	Adsorption
Basic oxygen furnace slag (BOFS)	Sorption processes
Resins of ion exchangers	Adsorption
Limestone products	Precipitation
Zero-valent iron (ZVI)	Reduction then precipitation
Apatite products	Precipitation
Sodium dithionite	Reduction and precipitation
Sulphate-reducing bacteria	Microbiological degradation
Zeolites products	Adsorption
Sand beds or gravel beds with nutrients and oxygen	Microbiological degradation

## Data Availability

The data presented in this study are included in the article. Further inquiries can be directed to the corresponding author.
